# Supplementary material for: Kinase inhibitor pulldown assay (KiP) for clinical proteomics
Source: Clin Proteomics. 2024 Jan 16;21:3. doi: 10.1186/s12014-023-09448-3 (PMC10790396; doi:10.1186/s12014-023-09448-3)
Supplement: Supplementary file 1 — Additional file 1: Figure S1. KiP with single inhibitor beads (sKiP). (A)Cartoon render of Abemaciclib in the binding pocket of target CDK4. Original RCSB PDB number 7SJ3, [39]. Note the cyclin was removed for this render. (B) Correlation matrix of the resultant kinome from each sKiP (single KiP) shows technical reproducibility of each sKiP (single KiP) across 3 different technicians. (C) Kinase family identified by sKiP. (D) Kinome Tree by sKiP. Illustration reproduced courtesy of Cell Signaling Technology, Inc. Colors are IDG kinase classifications. Green: Tbio, orange: Tchem, blue: Tclin, black: Tdark. (www.cellsignal.com). Drug abbreviations: ABE, abemaciclib; AFA, afatinib; AXI, axitinib; AZD, AZD4547; CRI, crizotinib; CZC, CZC-8004; FRX, FRAX597; PAL, palbociclib; GSK, GSK693693. Figure S2. KiP with different input experiment. (A) KiP was carried out with different amounts of lysate (Fig. 2A) and quantified kinase levels are plotted. Illuminating the Druggable Genome (IDG) Target Development Level (IDG-TDL) category indicated with different colors [26]. Green: Tbio, orange: Tchem, blue: Tclin, black: Tdark. FunCats are an in-house annotation of Functional Categories for different kinase targets including lipids (KI-L), metabolite (small molecule) (KI-M), proteins (KI-P) and unknown (KI-X). FunCats mapping table is available in the Additional file 2. (B-E) ABL1, ERBB2, CDK4 and PRKDC quantification was plotted as representative examples of linear and nonlinear responses. Figure S3. Detailed description of peptide assessment for PRM development. (A) Example of qPick from iSPEC database. All the information of identified peptides for each kinase in iSPEC database are presented by qPick. It includes peptide sequence, mass, gene product number for the peptide, miscleavage, PSMs for each modification, PSMs for each charge, best ion score, average retention time, etc. Peptides were ranked by experimental PSM counts, and top 3 to 6 peptides were selected for PRM runs. [file 12014_2023_9448_MOESM1_ESM.docx]

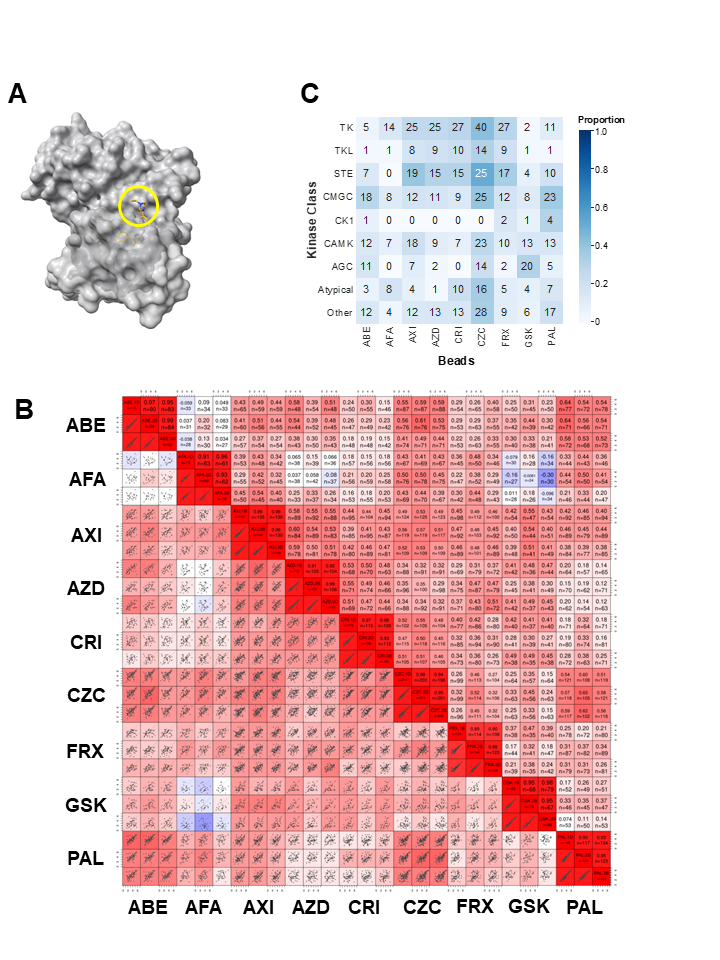


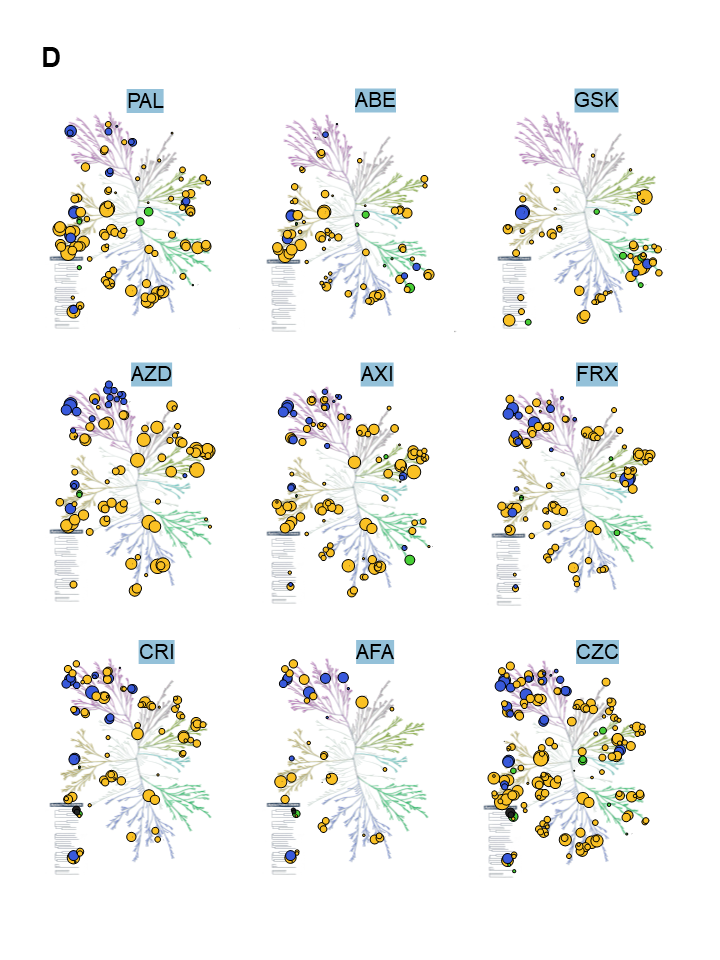


**Fig S1. KiP with single inhibitor beads (sKiP)**

1. Cartoon render of Abemaciclib in the binding pocket of target CDK4. Original RCSB PDB number 7SJ3, (39). Note the cyclin was removed for this render
2. Correlation matrix of the resultant kinome from each sKiP (single KiP) shows technical reproducibility of each sKiP (single KiP) across 3 different technicians.
3. Kinase family identified by sKiP
4. Kinome Tree by sKiP. Illustration reproduced courtesy of Cell Signaling Technology, Inc. Colors are IDG kinase classifications. Green: Tbio, orange: Tchem, blue: Tclin, black: Tdark. ([www.cellsignal.com](http://www.cellsignal.com)).

Drug abbreviations: ABE, abemaciclib; AFA, afatinib; AXI, axitinib; AZD, AZD4547; CRI, crizotinib; CZC, CZC-8004; FRX, FRAX597; PAL, palbociclib; GSK, GSK693693.


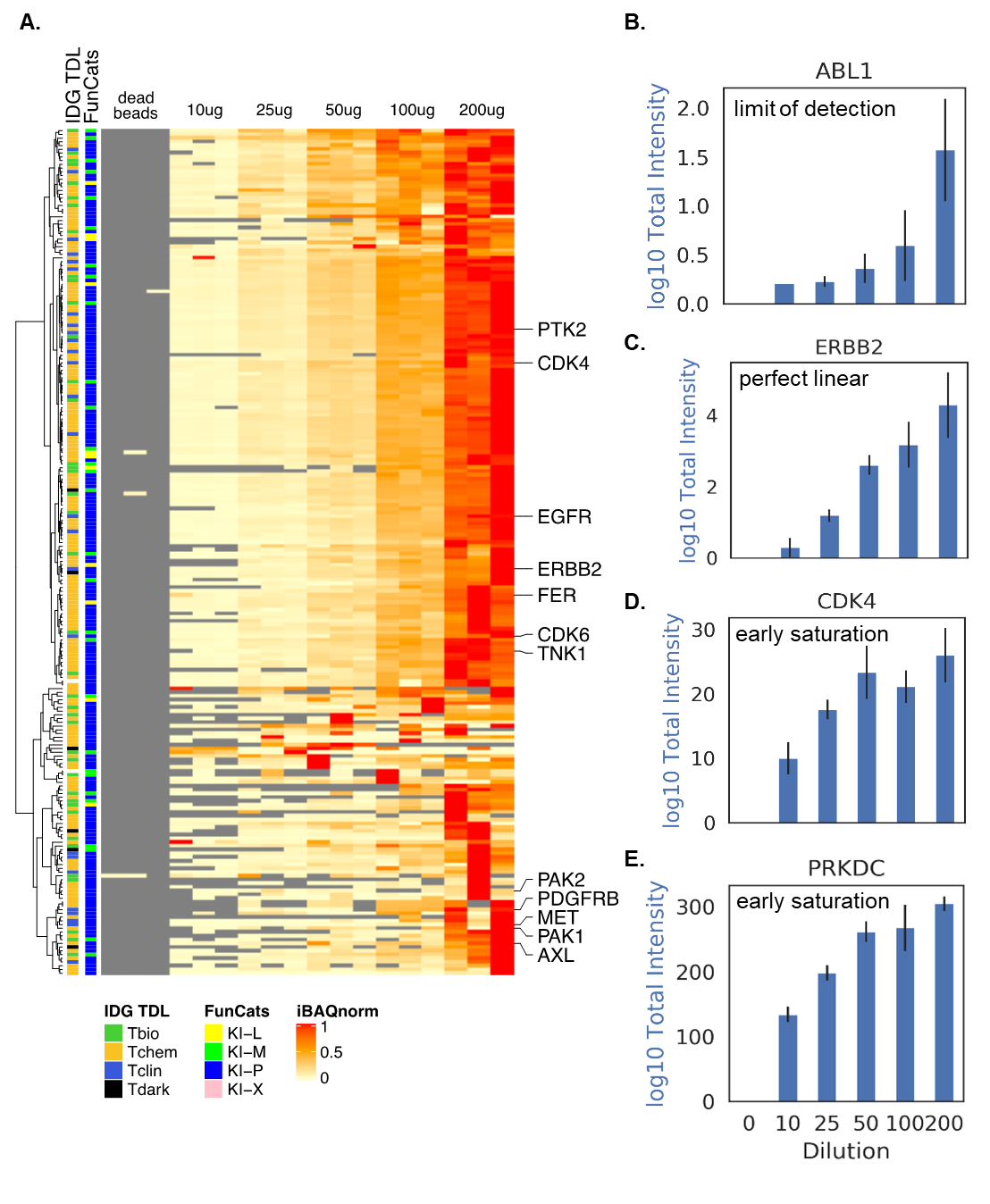


**Fig S2. KiP with different input experiment**

1. KiP was carried out with different amounts of lysate (Figure 2A) and quantified kinase levels are plotted. Illuminating the Druggable Genome (IDG) Target Development Level (IDG-TDL) category indicated with different colors (27). Green: Tbio, orange: Tchem, blue: Tclin, black: Tdark. FunCats are an in-house annotation of Functional Categories for different kinase targets including lipids (KI-L), metabolite (small molecule) (KI-M), proteins (KI-P) and unknown (KI-X). FunCats mapping table is available in the Supplementary Data.

(B-E) ABL1, ERBB2, CDK4 and PRKDC quantification was plotted as representative examples of linear and nonlinear responses.


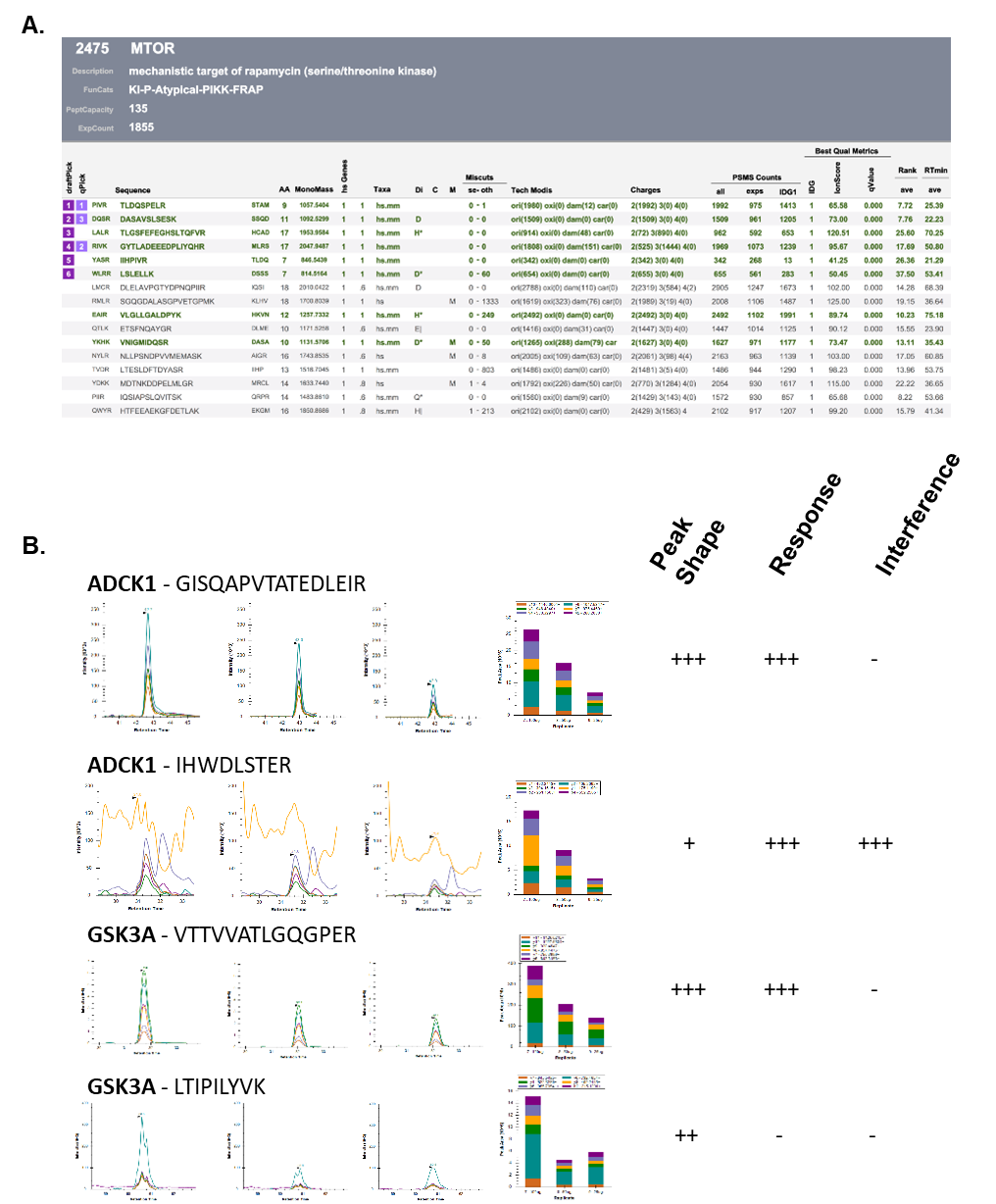


**Fig S3. Detailed description of peptide assessment for PRM development**

(A) Example of **qPick** from **iSPEC** database

All the information of identified peptides for each kinase in iSPEC database are presented by qPick. It includes peptide sequence, mass, gene product number for the peptide, miscleavage, PSMs for each modification, PSMs for each charge, best ion score, average retention time, etc.

Peptides were ranked by experimental PSM counts, and top 3 to 6 peptides were selected for PRM runs. However, peptides are excluded if they fall into following categories and other viable candidates exist; (1) More than 10% of PSMs has modification (2) A peptide has miscleavage in it (3) Many PSMs of that peptide are part of miscleaved peptides (4) Sequence is shared with other gene product (5) bad Mascot ion score (< 20).

(B) Representative examples of PRM peptide selection

KiP experiment was performed with different amounts of inputs and samples ran on mass spectrometry with PRM method to choose best PRM peptides. We took the following categories into consideration. (1) Peak shapes – peaks need to be symmetrical and narrow (2) response – sum of peak areas should be proportional to input level (3) interference – there should be no other non-specific peaks. We chose the peptides which meet these categories and generated the final list of PRM peptides for kinases. For example, peptide IHWDLSTER for ADCK1 has non-specific peaks around although peptide response looks good. On the other hand, peptide LTIPILYVK for GSK3A shows bad response whereas peak shape is good and there is no non-specific band. Therefore, these peptides were excluded from the final PRM peptide list.
